# Plasma oxytocin and leptin in relation to disordered eating: evidence from non-linear modeling across metabolic obesity phenotypes

**DOI:** 10.3389/fendo.2025.1693509

**Published:** 2025-11-20

**Authors:** Sevara Anvarova, Gulchekhra Narimova, Anna Aliyeva, Zamira Khalimova, Khurshida Nasirova

**Affiliations:** 1 Neuroendocrinology Department, Republican Specialized Scientific-Practical Medical Center of Endocrinology named after acad. Ya.Kh. Turakulov, Tashkent, Uzbekistan; 2 Diabetology Department, Republican Specialized Scientific-Practical Medical Center of Endocrinology named after acad. Ya.Kh. Turakulov, Tashkent, Uzbekistan; 3 Endocrinology Department, Tashkent State Medical University, Tashkent, Uzbekistan

**Keywords:** oxytocin, leptin, disordered eating, metabolic obesity phenotypes, spline modeling, restricted cubic splines, ROC curve analysis

## Abstract

**Background:**

Obesity is heterogeneous across metabolic and behavioral dimensions. Oxytocin, a hypothalamic neuropeptide, and leptin, an adiposity signal, have been implicated in appetite and reward, yet their relationships with disordered eating across metabolic obesity phenotypes remain unclear. We examined these associations and evaluated the predictive value of oxytocin alone versus multivariable models.

**Methods:**

In a cross-sectional cohort of 99 adults, we assessed anthropometry, biochemistry, oxytocin and leptin, and three validated questionnaires (EDE-Q, DEBQ, EBA-O). Participants were classified into four metabolic obesity phenotypes. Group differences used Kruskal–Wallis with Dunn’s correction; associations used Spearman correlation and OLS with HC3 robust SEs. Predictive modeling used logistic regression with restricted cubic splines for oxytocin and an elastic-net multivariable model (oxytocin spline + leptin, BMI, waist circumference, HSI, VAI, and a PCA-derived EDE-Q component). Performance was estimated via leakage-free nested cross-validation (outer 5-fold, inner 5-fold) using out-of-fold (OOF) ROC AUC, Brier score, bootstrap CIs, calibration, and decision-curve analysis.

**Results:**

Oxytocin was lower and leptin higher in metabolically unhealthy obesity (both p<0.01). Oxytocin correlated inversely with disordered-eating severity, while leptin correlated positively. The oxytocin-only spline model achieved OOF AUC 0.87 (95% CI 0.76–0.95; Brier 0.10). The combined elastic-net model achieved OOF AUC 0.97 (95% CI 0.90–1.00; Brier 0.05) and provided significantly better discrimination than oxytocin alone (ΔAUC 0.11, 95% CI 0.01–0.22; p=0.02). Using Youden’s index on OOF predictions, the oxytocin-only model’s optimal operating probability (0.69) mapped to ~90.5 pg/mL (95% CI 74.8–103.3), yielding sensitivity of 0.94 (0.87–0.99) and specificity of 0.83 (0.70–0.95). Decision-curve analysis showed higher net benefit for multivariable models across clinically relevant thresholds.

**Conclusion:**

Lower oxytocin is associated with greater disordered-eating severity, but oxytocin is most informative when integrated with metabolic and behavioral markers. A multivariable model substantially improved discrimination and net benefit over oxytocin alone. The ~90.5 pg/mL value is an exploratory operating point rather than a clinical cutoff; external validation and prospective evaluation are needed before translation to practice.

## Introduction

Obesity and its metabolic complications are a growing global health crisis, contributing substantially to cardiovascular disease, type 2 diabetes, and non-alcoholic fatty liver disease (NAFLD) (1). Although body mass index (BMI) remains the most common clinical indicator, obesity is heterogeneous: some individuals with elevated BMI are metabolically healthy, whereas others with normal weight show marked metabolic dysfunction. This heterogeneity underscores the need to assess obesity through both metabolic and behavioral dimensions (2).

Disordered eating patterns—binge eating, emotional eating, external eating, night eating—are key behavioral contributors to excess energy intake and weight gain but are often under-recognized in routine care (3). Psychometric tools such as the Eating Disorder Examination Questionnaire (EDEQ), Dutch Eating Behavior Questionnaire (DEBQ), and the Eating Behavior Assessment for Obesity (EBA-O) can profile these behaviors in clinical and research settings (4–6).

Neuroendocrine pathways are increasingly implicated in disordered eating. Oxytocin—a hypothalamic neuropeptide involved in social bonding, stress responsivity, and energy balance—has been shown to modulate appetite, attenuate reward-driven intake, and enhance cognitive control (7). Preclinical work suggests oxytocin reduces caloric intake and body weight, especially under high-fat diet or stress conditions (8). Early clinical studies indicate intranasal oxytocin may lower energy intake, improve insulin sensitivity, and reduce BMI in obesity and binge-eating populations (9). In parallel, leptin—an adiposity signal often elevated in obesity—has been linked to reward-related eating and putative leptin resistance (10).

However, relationships between endogenous circulating oxytocin, metabolic health, and disordered eating remain incompletely characterized, particularly across distinct metabolic obesity phenotypes (metabolically healthy normal weight [MHNW], metabolically unhealthy normal weight [MUNW], metabolically unhealthy overweight [MUOW], and metabolically unhealthy obese [MUO]). Prior studies often treat obesity as homogeneous, rarely test non-linear oxytocin–risk relationships, and seldom evaluate whether oxytocin adds predictive value beyond metabolic and behavioral measures. Data from Central Asia are especially scarce despite rising obesity prevalence; for example, recent reports from Uzbekistan indicate substantial increases in adult and adolescent obesity with implications for cardiometabolic burden: national obesity rates have risen dramatically in recent decades, with 27.9% of adult men and approximately 5.7% of adolescents now classified as obese [NCD-RisC, 2024]. The 2025 World Obesity Atlas projects further increases in prevalence, with implications for cardiometabolic disease burden and healthcare costs (11).

In this context, the present study investigates associations between plasma oxytocin levels, metabolic obesity markers, and eating behavior traits across a spectrum of metabolic obesity phenotypes in adults. We compared oxytocin and leptin concentrations across the four predefined metabolic obesity phenotypes (MHNW, MUNW, MUOW, and MUO). We assessed the prevalence and severity of disordered eating using three validated questionnaires (EDE-Q, DEBQ, and EBA-O) and examined correlations between metabolic/hormonal parameters and disordered eating behaviors. Additionally, we identified independent predictors of insulin resistance and eating disorder severity through multiple linear regression analysis. We also explored an operating point for oxytocin derived from out-of-fold predictions, recognizing this is not a clinical cutoff.

By integrating anthropometric, biochemical, and psychometric data, this study offers a comprehensive view of the metabolic and neuroendocrine underpinnings of obesity and disordered eating, and provides novel insights into the potential therapeutic role of oxytocin.

## Materials and methods

This cross-sectional, observational study was conducted at the Republican Specialized Scientific-Practical Medical Center of Endocrinology named after Academician Ya. Kh. Turakulov (Tashkent, Uzbekistan) between March and June 2025. The design followed the STROBE recommendations for cross-sectional research (**Supplementary Table S1**). Adults aged 18–65 years with a body mass index (BMI) ≥ 18.5 kg/m² and without acute or chronic systemic disease were eligible. Exclusion criteria included pregnancy or lactation, psychiatric or neurological disorders, pharmacologic treatment affecting appetite or metabolism (e.g., corticosteroids, antidepressants), diabetes mellitus or other endocrine disease, and inability to complete study procedures. The study protocol was reviewed and approved by the Local Bioethics Committee of the Republican Specialized Scientific-Practical Medical Center of Endocrinology named after Academician Turakulov (Protocol No. 2/2025, dated 12 February 2025). All procedures were performed in accordance with the ethical principles outlined in the Declaration of Helsinki (2013 revision). Written informed consent was obtained from all participants prior to enrollment.

### Anthropometric and clinical evaluation

Measurements were obtained between 08:00 and 10:00 a.m. after an overnight fast and bladder emptying. Body weight and height were measured using a calibrated Seca^®^ scale and stadiometer, and BMI was calculated as weight (kg)/height² (m²). Waist circumference (WC) was measured midway between the lower rib and the iliac crest, hip circumference (HC) at the widest part of the buttocks, and waist-to-hip ratio (WHR) as WC/HC.

### Biochemical and hormonal analyses

After a fasting period ≥ 10 h, venous blood was collected between 08:00 and 09:00 a.m. Plasma oxytocin and leptin concentrations were determined by competitive and sandwich enzyme-linked immunosorbent assays (ELISA; Elabscience^®^, Wuhan, China). Pre-analytical handling and assay calibration followed standardized protocols to minimize variability (see **Supplementary Methods** for full assay specifications and quality-control parameters). Standard biochemical markers (glucose, HbA1c, lipid profile, alanine and aspartate aminotransferases [ALT, AST], and gammaglutamyltransferase [GGT]) were analyzed on a HITACHI 902 automated analyzer; serum insulin was measured by electrochemiluminescence on a COBAS e 411 analyzer (Roche Diagnostics, Germany).

Derived indices included the Homeostatic Model Assessment of Insulin Resistance (HOMAIR) (12), Hepatic Steatosis Index (HSI) (13), and Atherogenic Index (AI). The Visceral Adiposity Index (VAI) was calculated using sex-specific equations incorporating WC, BMI, triglycerides (TG), and HDL-cholesterol (HDL-C) (14).

### Behavioral assessment

Eating behavior was evaluated using three validated self-report instruments:

1. the Eating Disorder Examination Questionnaire (EDE-Q 6.0), evaluating restraint, eating concern, shape concern, and weight concern, with a global score > 2.3 indicating clinical significance (4).
2. the Dutch Eating Behavior Questionnaire (DEBQ), assessing emotional, external, and restrained eating (high-risk thresholds > 3.25 for emotional and > 3.5 for external eating; α = 0.91–0.95) (5); and
3. Eating Behavior Assessment for Obesity (EBA-O), which screens for food-addiction, binge-, night, sweet-, and hyperphagic eating patterns (cut-off ≥ 4 indicates pathology; Cronbach’s α = 0.88) (6);

In this study, we focus on disordered eating behaviours (DEBs) — defined as sub-clinical or behavioural manifestations of eating attitudes/behaviours — rather than categorical DSM-5 eating disorder diagnoses. Participants completed questionnaires independently under supervision of trained staff, and a clinical psychologist reviewed responses when indicated.

### Metabolic phenotyping

Participants were classified into four metabolic obesity phenotypes (MHNW, MUNW, MUOW, MUO) according to BMI, HOMA-IR, and HSI criteria. Cut-off definitions and rationale are presented in **Supplementary Methods** (Phenotyping Definitions section).

These phenotype definitions enabled a more precise investigation of how plasma oxytocin levels relate to obesity, metabolic dysfunction, and eating behavior beyond BMI alone.

### Statistical analyses

All statistical analyses were conducted in Python 3.11 using Pandas, NumPy, SciPy, Seaborn, Statsmodels, and Scikit-learn libraries. Descriptive statistics were calculated for all variables.

Continuous variables are presented as mean ± standard deviation (SD) or median (interquartile range, IQR) according to distribution; categorical variables are presented as frequency and percentage. Normality was assessed using the Shapiro–Wilk test.

For between-group comparisons among the four metabolic obesity phenotypes (MHNW, MUNW, MUOW, MUO), the Kruskal–Wallis test was applied to continuous variables, followed by Dunn’s *post hoc* test with Bonferroni correction. Spearman correlation analysis was used to assess associations between plasma oxytocin levels and key metabolic markers (HOMA-IR, VAI, HSI), as well as eating behavior questionnaire scores (EDE-Q, DEBQ, EBA-O).

Multivariable models (ordinary least squares and elastic-net logistic regression) identified independent predictors of insulin resistance and disordered eating. All primary models were adjusted for age and sex, with sex × oxytocin interactions tested. Model performance was evaluated via nested cross-validation, receiver operating characteristic (AUC), calibration, and decision-curve analysis. Detailed specifications of model A and B, variable transformations, and hyperparameter tuning are reported in **Supplementary Methods** (Statistical Procedures section).

All main regression models were adjusted for age and sex, and effect modification by sex was assessed by including sex × oxytocin interaction terms.

Menstrual phase and hormonal contraceptive use—potentially relevant modifiers of oxytocin levels—were considered in the study design; however, these data were not available in the present cohort and are acknowledged as a limitation. Where available, additional potential confounders were included in sensitivity analyses: use of antidiabetic medications (metformin, GLP-1 receptor agonists), smoking status, alcohol intake, and average nightly sleep duration.

All tests were two-tailed, and p-values < 0.05 were considered statistically significant.

## Results

### Participant characteristics

The study included 99 participants (mean age 38.94 ± 10.40 years; 76.8% female). Median BMI was 32.10 kg/m² (IQR: 23.35–38.10), with a median body fat percentage of 36.90% (IQR: 23.38– 46.61). Central adiposity was indicated by waist circumference (96.87 ± 22.59 cm) and waist-to-hip ratio (0.83 ± 0.11).

Biochemical profiles revealed median ALT, AST, and GGT levels of 21.00, 21.00, and 36.00 U/L, respectively. Median fasting glucose was 5.10 mmol/L (IQR: 4.73–5.84) and HbA1c 5.10% (IQR: 4.70–5.70). The lipid profile showed median total cholesterol 4.81 mmol/L (IQR: 4.10–5.80), HDL-C 1.17 mmol/L, LDL-C 2.15 mmol/L, and triglycerides 1.15 mmol/L. Median atherogenic index was 3.03.

HOMA-IR was elevated (median 3.51, IQR: 1.68–5.34), alongside increased hepatic steatosis index (HSI: 43.96, IQR: 32.59–52.34) and visceral adiposity index (VAI: 3.43, IQR: 1.44–4.81). Hormonal analysis revealed hyperleptinemia and high inter-individual variability in oxytocin (median 37.60 pg/mL, IQR: 22.30–122.35).

Eating behavior assessments indicated high scores in weight concern, shape concern, and restrained eating (EDE-Q) as well as binge eating and sweet eating (EBA-O). Most variables were nonnormally distributed (Shapiro–Wilk p < 0.05) (**Table 1**).

**Table 1:** Baseline characteristics of study participants.

Category	Variable	Value	Shapiro–Wilk p
Demographic & Anthropometric	Age (years)	38.94 ± 10.40	0.3362
	BMI (kg/m²)	32.10 (23.35–38.10)	0.0001
	Body fat (%)	36.90 (23.38–46.61)	0.0101
	Height (cm)	164.00 (159.50–168.00)	0.000
	Hip circumference (cm)	114.00 (99.00–130.00)	0.0447
	Waist circumference (cm)	96.87 ± 22.59	0.0525
	Waist-to-hip ratio	0.83 ± 0.11	0.1861
	Weight (kg)	86.00 (64.50–105.00)	0.000
Biochemical analyses	ALT (U/L)	21.00 (16.85–31.85)	0.000
	AST (U/L)	21.00 (18.00–27.60)	0.000
	GGT (U/L)	36.00 (20.00–54.15)	0.000
	Glucose (mmol/L)	5.10 (4.73–5.84)	0.000
	HbA1c (%)	5.10 (4.70–5.70)	0.000
	Total cholesterol mmol/L	4.81 (4.10–5.80)	0.0023
	HDL-C mmol/L	1.17 (1.00–1.42)	0.0001
	LDL-C mmol/L	2.15 (1.90–3.01)	0.000
	Triglycerides (mmol/L)	1.15 (0.80–2.07)	0.000
Indices	HOMA-IR	3.51 (1.68–5.34)	0.000
	Hepatic Steatosis Index (HSI)	43.96 (32.59–52.34)	0.0003
	Visceral Adiposity Index (VAI)	3.43 (1.44–4.81)	0.000
	Atherogenic index	3.03 (2.55–3.56)	0.000
Hormonal analyses	Insulin (μU/mL)	13.18 (7.20–21.61)	0.000
	Leptin (pg/mL)	1157.10 (416.95–1180.25)	0.000
	Oxytocin (pg/mL)	37.60 (22.30–122.35)	0.000
DEBQ Subscales	Emotional Eating (EE-score)	1.46 (0.60–2.48)	0.000
	External Eating (EX-score)	2.50 (1.00–3.20)	0.000
	Restrained Eating (RE-score)	2.40 (1.30–3.30)	0.000
EBA-O Subscales	Binge Eating (BEscore)	1.50 (0.30–4.00)	0.000
	Food Addiction (FA-score)	0.66 (0.20–2.00)	0.000
	Hyperphagia (HP-score)	1.00 (0.30–2.00)	0.000
	Night Eating (NEscore)	0.00 (0.00–1.00)	0.000
	Sweet Eating (SEscore)	0.80 (0.70–3.90)	0.000
EDE-Q Subscales	Binge Eating Frequency (episodes)	0.50 (0.00–1.42)	0.000
	Eating Concern (EC-score)	2.00 (0.60–3.58)	0.000
	Global EDE-Q	3.39 (1.00–3.87)	0.000
	Restraint (Re-score)	1.20 (0.75–2.80)	0.000
	Shape Concern (SCscore)	4.00 (2.80–4.66)	0.000
	Weight Concern (WC-score)	4.50 (1.80–5.78)	0.000

Data are presented as mean ± SD or median (IQR) according to distribution; p-values from Shapiro–Wilk test are shown. Variables are grouped by category.

### Group comparisons of metabolic and hormonal parameters

Participants were stratified into four metabolic obesity phenotypes: MHNW (n = 18), MUNW (n = 12), MUOW (n = 13), and MUO (n = 56). Kruskal–Wallis tests indicated significant between-group differences in plasma oxytocin (H = 25.675, p < 0.001) and leptin (H = 46.225, p < 0.001).

**Figure 1:**
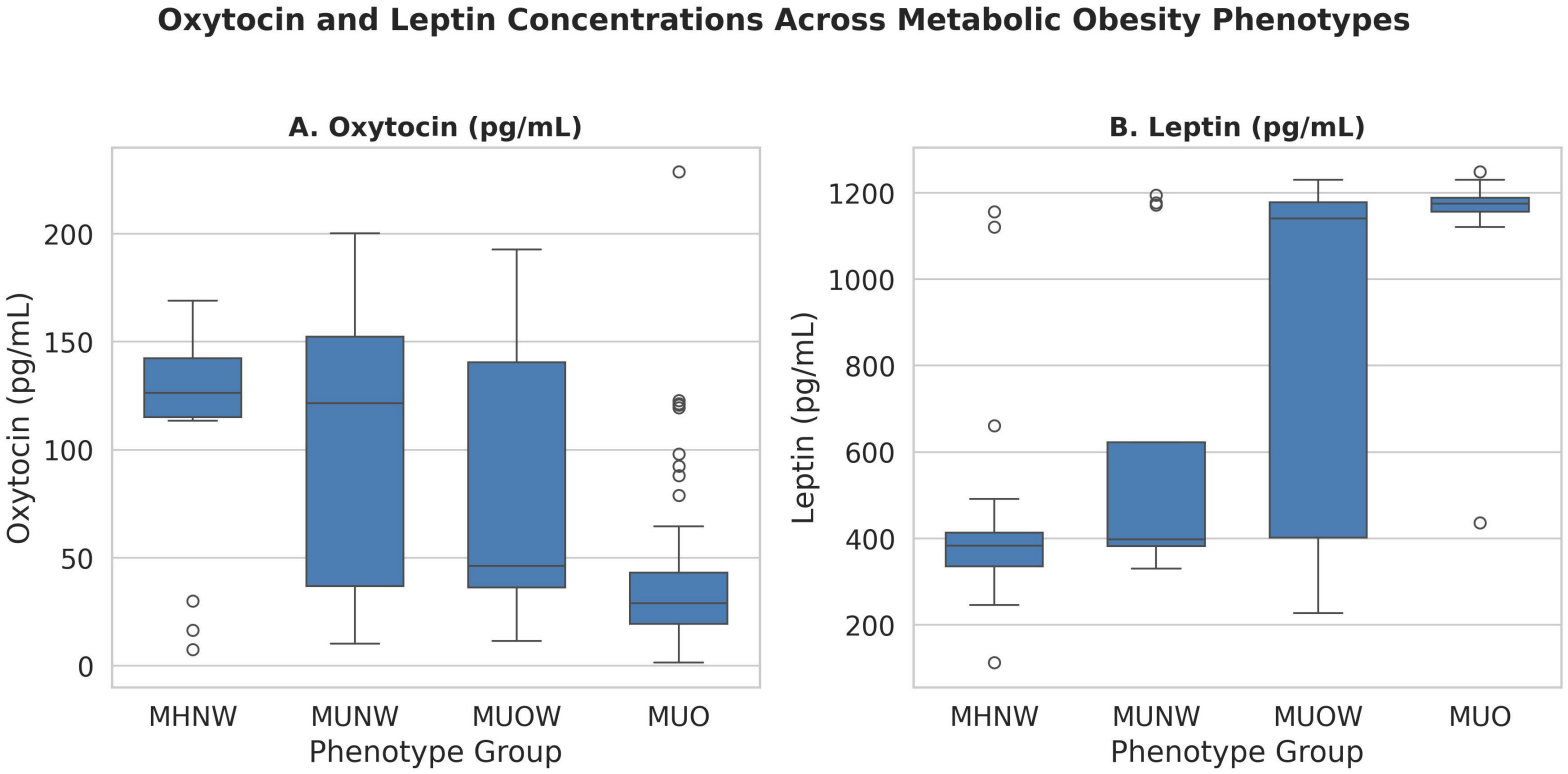
Oxytocin and leptin concentrations across metabolic obesity phenotypes. Data are shown as boxplots with medians and interquartile ranges. Kruskal–Wallis p-values are reported; asterisks indicate significant pairwise differences (Dunn’s test with FDR adjustment). MHNW, Metabolically healthy normal Weight; MUNW, Metabolically unhealthy normal weight; MUOW, Metabolically unhealthy overweight MUO, Metabolically unhealthy obesity.

*Post hoc* Dunn’s tests showed that MUO had significantly lower oxytocin compared to MHNW (p < 0.0001), MUNW (p = 0.014), and MUOW (p = 0.037), and significantly higher leptin than all other phenotypes (p < 0.001) (**Figure 1**).

### Prevalence of disordered eating behaviors

Based on validated cut-off scores, the most prevalent disordered eating patterns were Weight Concern (57.6%) and Shape Concern (54.5%) on the EDE-Q, followed by Binge Eating (26.3%) and Sweet Eating (25.3%) on the EBA-O. Less common behaviors included Hyperphagia (7.1%), Food Addiction (4.0%), and Night Eating (3.0%). In the DEBQ subscales, Restrained Eating was present in 23.2% of participants, External Eating in 18.2%, and Emotional Eating in 10.1% (**Table 2**).

**Table 2:** Correlations between hormonal, metabolic, and behavioral variables.

Questionnaire	Subscale	Cut-off	Disordered (≥ cut-off)	% Disordered
EDE-Q	Weight Concern (WC)	≥ 4.0	57	57.6%
	Shape Concern (SC)	≥ 4.0	54	54.5%
	Eating Concern (EC)	≥ 4.0	19	19.2%
	Dietary Restraint (DR)	≥ 4.0	6	6.1%
EBA-O	Binge Eating (BE)	≥ 4.0	26	26.3%
	Sweet Eating (SE)	≥ 4.0	25	25.3%
	Hyperphagia (HP)	≥ 4.0	7	7.1%
	Food Addiction (FA)	≥ 4.0	4	4.0%
Questionnaire	Subscale	Cut-off	Disordered (≥ cut-off)	% Disordered
	Night Eating (NE)	≥ 4.0	3	3.0%
DEBQ	Restrained Eating (RE)	≥ 3.5	23	23.2%
	External Eating (EX)	≥ 3.5	18	18.2%
	Emotional Eating (EE)	≥ 3.5	10	10.1%

Data are shown as absolute numbers (n) and percentages of participants meeting the cut-off criteria.

### Disordered eating patterns across metabolic obesity phenotypes

**Figure 2:**
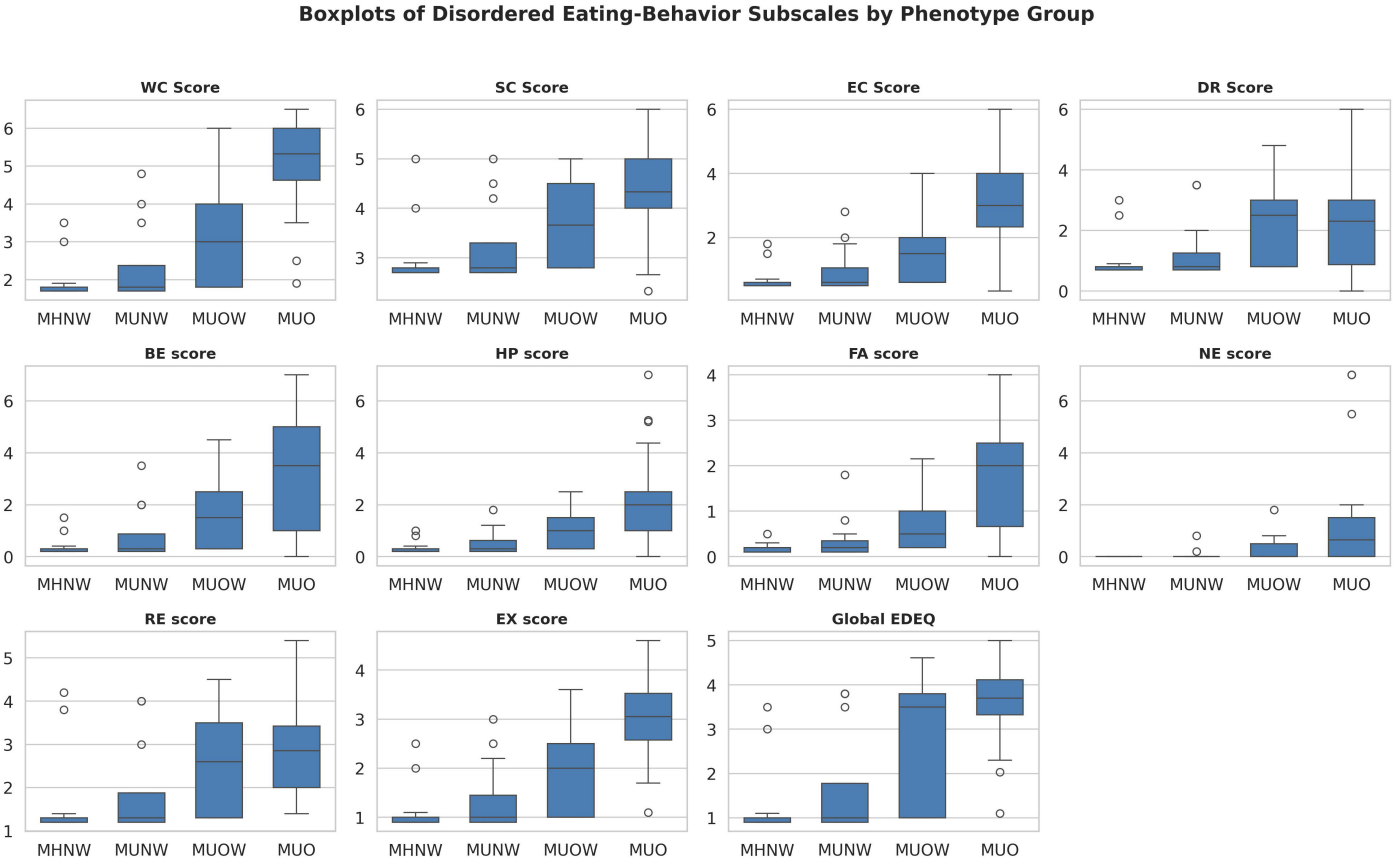
Boxplots of significant disordered eating subscales by metabolic obesity phenotype. Scores are shown for Weight Concern (WC), Shape Concern (SC), Eating Concern (EC), Dietary Restraint (DR), Binge Eating (BE), Hyperphagia (HP), Food Addiction (FA), Night Eating (NE), Restrained Eating (RE), External Eating (EX), and global Eating Disorder Examination Questionnaire (EDE-Q). Groups: MHNW — metabolically healthy normal weight; MUNW — metabolically unhealthy normal weight. MUOW — metabolically unhealthy overweight; MUO — metabolically unhealthy obese; Horizontal lines within boxes indicate medians; boxes denote interquartile range (IQR); whiskers indicate 1.5 × IQR; circles indicate outliers. Kruskal–Wallis p-values were controlled for false discovery rate (FDR < 0.05), with *post hoc* Dunn’s test for pairwise comparisons.

Kruskal–Wallis tests indicated differences in weight concern, shape concern, eating concern, restraint, binge eating, hyperphagia, food addiction, night eating, and global EDE-Q (all p<0.05 after FDR). Sweet eating was non-significant (q=0.13). Dunn’s tests (Bonferroni-adjusted) showed MUO with higher global EDE-Q than MHNW and MUNW (median difference +1.8, Cohen’s d≈1.0; p<0.01). MUO differed from MUOW in select subscales (e.g., weight concern, eating concern). MHNW and MUNW showed no differences (**Supplementary Table 2**; **Figure 2**).

### Correlation analysis

**Figure 3:**
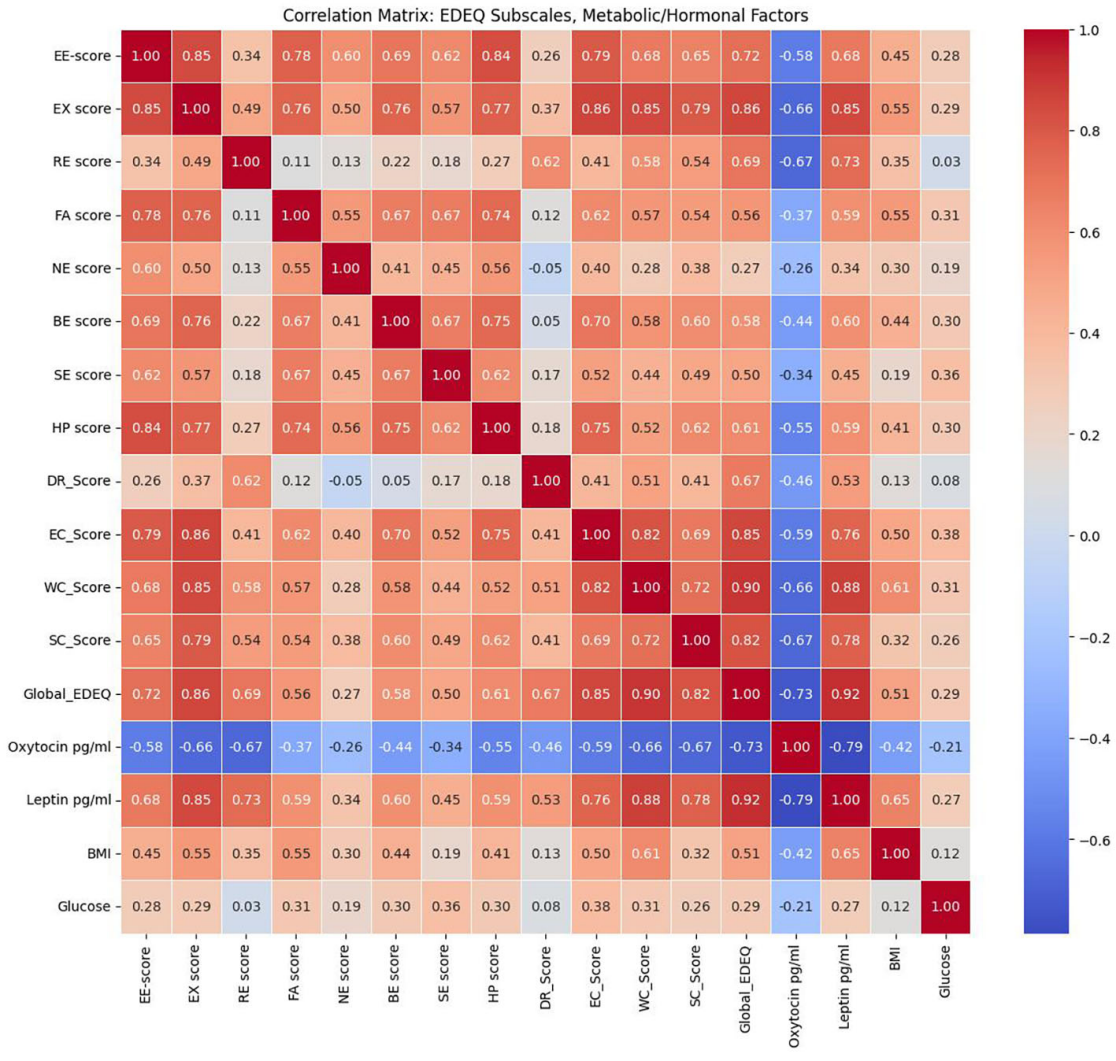
Heatmap of Spearman correlation coefficients between metabolic/hormonal parameters and eating behavior scores.

A heatmap of Spearman correlation coefficients (**Figure 3**) revealed strong intercorrelations among the EDE-Q subscales and the Global EDE-Q score, reflecting the internal consistency of this instrument. Notably, Global EDE-Q demonstrated strong positive correlations with EE-score, EX score, RE score, EC_Score, WC_Score, and SC_Score.

Significant associations with metabolic and hormonal variables were also observed. Leptin levels showed robust positive correlations with the majority of EDE-Q subscales and the global score, indicating a relationship between higher leptin concentrations and greater disordered eating severity. In contrast, oxytocin levels were inversely correlated with nearly all EDE-Q domains, suggesting a potential protective or regulatory role. BMI and glucose had modest positive associations (r≈0.30–0.45, p<0.05). HOMA-IR and HSI correlated moderately (r≈0.52, p<0.001). NAFLD risk (HSI ≥36) was 78% in MUO vs. 11% in MHNW (p<0.001) and correlated with EDE-Q global scores (r=0.41, p<0.01) (**Table 3**).

**Table 3:** Regression models predicting HOMA-IR and EDE-Q Global score.

EDE-Q Variable	Oxytocin (pg/mL)	Leptin (ng/mL)	BMI (kg/m²)	Glucose (mmol/L)
EE-score	r = –0.582 p < 0.001	r = 0.684 p < 0.001	r = 0.450 p < 0.001	r = 0.278 p = 0.006
EX score	r = –0.665 p < 0.001	r = 0.847 p < 0.001	r = 0.549 p < 0.001	r = 0.291 p = 0.004
RE score	r = –0.669 p < 0.001	r = 0.733 p < 0.001	r = 0.355 p < 0.001	r = 0.355 p < 0.001
FA score	r = –0.368 p < 0.001	r = 0.593 p < 0.001	r = 0.551 p < 0.001	r = 0.312 p = 0.002
NE score	r = –0.261 p = 0.010	r = 0.336 p = 0.001	r = 0.298 p = 0.003	—
BE score	r = –0.442 p < 0.001	r = 0.601 p < 0.001	r = 0.443 p < 0.001	r = 0.296 p = 0.003
SE score	r = –0.344 p = 0.001	r = 0.451 p < 0.001	—	r = 0.364 p < 0.001
HP score	r = –0.551 p < 0.001	r = 0.591 p < 0.001	r = 0.414 p < 0.001	r = 0.297 p = 0.003
EC Score	r = –0.585 p < 0.001	r = 0.761 p < 0.001	r = 0.496 p < 0.001	r = 0.380 p < 0.001
WC Score	r = –0.662 p < 0.001	r = 0.880 p < 0.001	r = 0.612 p < 0.001	r = 0.310 p = 0.002
SC Score	r = –0.667 p < 0.001	r = 0.776 p < 0.001	r = 0.325 p = 0.001	r = 0.262 p = 0.010
Global EDE-Q	r = –0.734 p < 0.001	r = 0.919 p < 0.001	r = 0.505 p < 0.001	r = 0.290 p = 0.004

These results, visualized in the heatmap, highlight consistent associations between metabolic/hormonal dysregulation and the severity of disordered eating behaviors (**Figure 3**).

### Multiple regression analysis

Ordinary least squares models with HC3 robust standard errors were used to identify independent predictors of insulin resistance (HOMA-IR) and disordered eating severity (global EDE-Q). Candidate predictors included plasma oxytocin, leptin, body mass index (BMI), visceral adiposity index (VAI), and eating behavior subscales (emotional, external, restrained, food addiction, night eating, binge eating, sweet eating, and hyperphagia). Predictors were retained using backward elimination with a pre-specified threshold of p < 0.10, and model assumptions were verified through residual diagnostics (**Table 4**).

Table 4Summary of Bayesian modeling performance metrics.

**Table:** 

Model A — HOMA-IR (R²=0.52; adj R²=0.50; N = 99; all VIF<5).					
Predictor	B	95% CI (lower)	95% CI (upper)	β (standardized)	p-value
Intercept	-1.8766	-2.6823	-1.0709	—	<0.01
Leptin (pg/mL)	0.0027	0.0006	0.0048	0.337	0.01
BMI	0.1385	0.0979	0.179	0.468	<0.01
(kg/m²)					
RE score	-0.7255	-1.4371	-0.0139	-0.27	0.05
SE score	0.3075	0.0664	0.5486	0.221	0.01

**Table:** 

Model B — Global EDE-Q (R²=0.904; adj R²=0.896; N = 99; Max VIF ≈ 8.46 for EX)					
Predictor	B	95% CI (lower)	95% CI (upper)	β (standardized)	p-value
Intercept	-0.5367	-0.7329	-0.3404	—	<0.01
Leptin (pg/mL)	0.0023	0.0017	0.0029	0.626	<0.01
EE score	0.2286	-0.0207	0.478	0.183	0.07
EX score	0.5185	0.1422	0.8948	0.411	<0.01
FA score	-0.2193	-0.3805	-0.0582	-0.18	<0.01
NE score	-0.1877	-0.3233	-0.0521	-0.163	<0.01
BE score	-0.0996	-0.2035	0.0042	-0.154	0.06
SE score	0.1043	0.0273	0.1813	0.163	<0.01

Methods: OLS with HC3 robust SE; backward elimination threshold p=0.10. Coefficients (B) with 511 95% CI, standardized β, and p-values (two decimals).

For HOMA-IR, the model explained 52% of variance (adjusted R² = 0.50, F p < 0.01, VIF range 1.1– 3.5). Higher BMI, leptin, and sweet-eating scores were associated with higher insulin resistance, whereas restrained eating was inversely related.

For the global EDE-Q score, the model explained 90% of variance (adjusted R² = 0.90, F p < 0.01, VIF < 4.0 except for external eating ≈ 8.5). Higher leptin, external eating, and sweet eating were linked to more severe disordered eating, while food addiction and night eating showed negative associations.

A sensitivity analysis using principal component analysis (PCA) to replace correlated subscales reduced multicollinearity (maximum VIF ≈ 4.9) and yielded comparable estimates, confirming the stability of the main results.

### Predictive modeling of disordered eating behavior

We evaluated oxytocin and related metabolic and behavioral variables to predict elevated disordered eating (Global EDE-Q ≥ 2.5) using a leakage-free nested 5×5 cross-validation framework. All preprocessing (imputation, scaling, spline transformation for oxytocin, and PCA of EDE-Q subscales) and elastic-net hyperparameter tuning were performed within folds.

**Figure 4:**
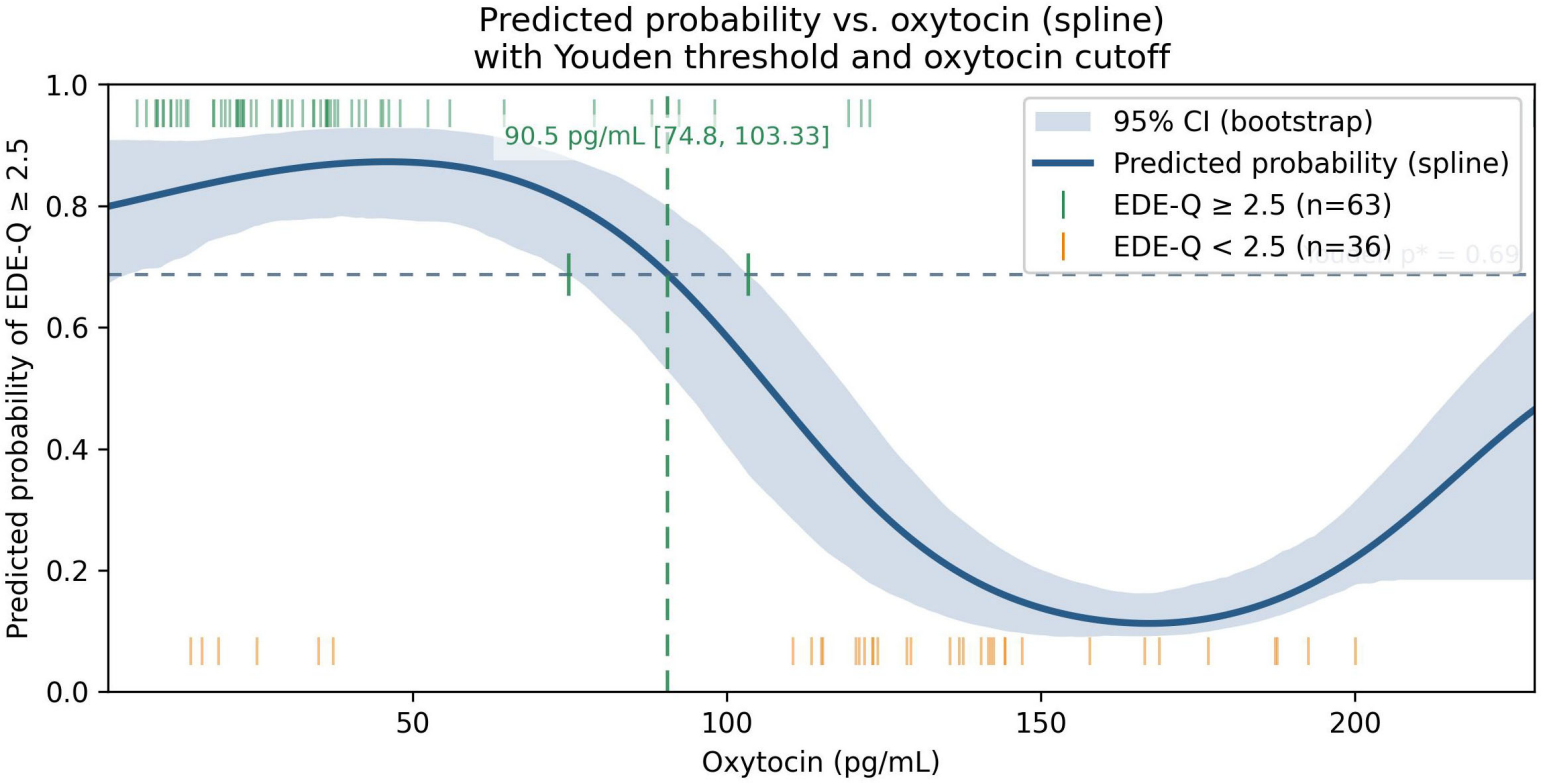
Predicted probability of EDE-Q ≥ 2.5 as a function of plasma oxytocin from a restrictedcubic-spline logistic model (solid line) with 95% bootstrap CI (shaded). The horizontal dotted line shows the Youden-optimal probability threshold (p* = 0.69); the vertical dashed line shows the corresponding oxytocin cutoff (90.5 pg/mL, 95% CI 74.8–103.3). Rug ticks denote observed positives (top) and negatives (bottom). Threshold and CI were derived from out-of-fold predictions and bootstrap mapping, respectively. the Youden threshold and its mapped oxytocin value represent an exploratory operating point derived from out-of-fold predictions; they are not intended as a clinical decision cutoff and would require external validation and prospective evaluation.

The oxytocin-only spline model achieved an AUC of 0.87 (95% CI 0.76–0.95) and a mean Brier score of 0.10, with an optimal threshold corresponding to an oxytocin concentration of approximately 90.5 pg/mL (**Figure 4**).

**Figure 5:**
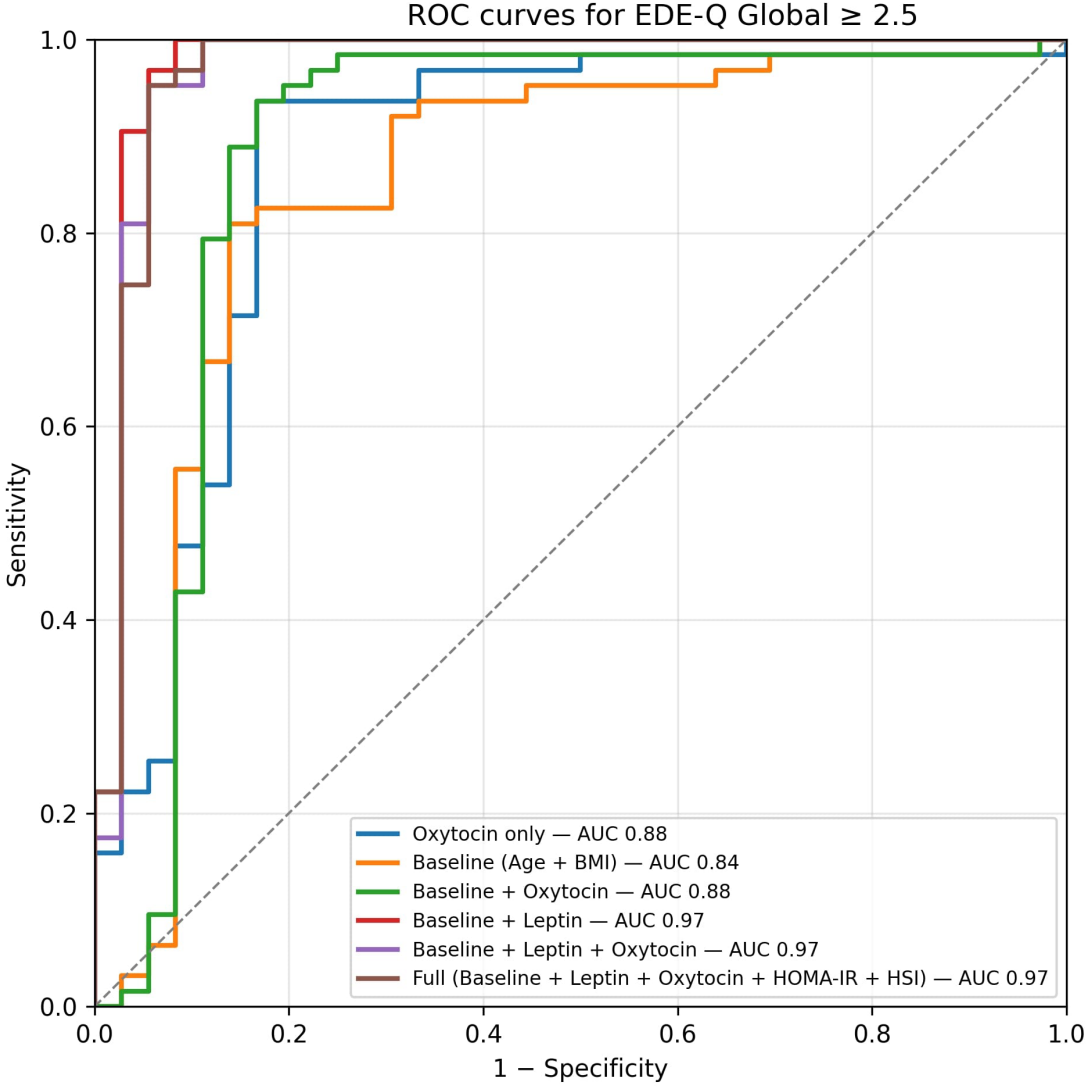
ROC curves from nested cross-validation (OOF). The oxytocin-only spline model achieved AUC 0.87 (95% CI 0.76–0.95), Brier 0.10; the combined model achieved AUC 0.97 (95% CI 0.90– 1.00), Brier 0.05. Dots mark Youden-optimal operating points (oxytocin-only: pˆ * 0.69, sensitivity 0.94, specificity 0.83; combined: pˆ * 0.77, sensitivity 0.98, specificity 0.92). Curves and metrics are based on OOF predictions. Dots mark the Youden-optimal operating points with 95% CIs (bootstrap, OOF): oxytocin-only — sens 0.94 (0.87–0.99), spec 0.83 (0.70–0.95); combined — sens 0.98 (0.95–1.00), spec 0.92 (0.82–1.00).”.

The multivariable elastic-net model, which included oxytocin spline, leptin, BMI, waist circumference, HSI, VAI, and a PCA-derived EDE-Q component, achieved AUC = 0.97 (95% CI 0.90– 1.00) and Brier = 0.05, significantly improving discrimination compared with the oxytocin-only model (ΔAUC = 0.11, 95% CI 0.01–0.22; *p* = 0.02; **Supplementary Tables S3**, **S4**; **Figure 5**).

**Figure 6:**
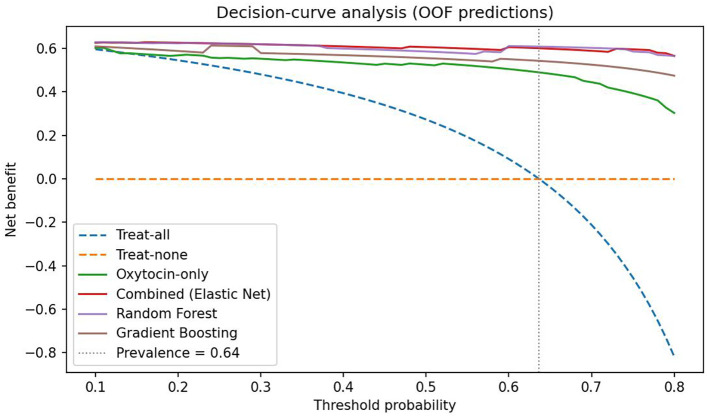
Decision-curve analysis based on out-of-fold predictions. Net benefit is plotted against threshold probability for classifying Global EDE-Q ≥ 2.5. The combined multivariable model (Elastic-Net) and Random Forest deliver the highest net benefit across 0.10–0.80, with Gradient Boosting close behind; the oxytocin-only spline model provides lower benefit at higher thresholds. “Treat-all” and “treat-none” strategies are shown for reference. The vertical dotted line marks the cohort prevalence (0.64). The red dot marks the Youden-optimal operating point for the combined model (p* = 0.77 from the OOF ROC), where sensitivity and specificity are jointly maximized.

Random Forest and Gradient Boosting models demonstrated comparable AUCs but poorer calibration slopes. Decision-curve analysis (**Figure 6**) showed that the combined model provided the highest net clinical benefit across threshold probabilities (0.10–0.80). At the cohort prevalence (0.64) and the Youden-optimal threshold (0.77), the combined model maintained a clearly positive net benefit, supporting its potential clinical utility for identifying individuals at elevated risk of disordered eating.

Bayesian hierarchical sensitivity analysis confirmed the stability of the principal associations across alternative prior strengths and likelihood formulations. Posterior estimates for oxytocin (β ≈ 0.9, 95% CrI: 0.75–1.04) and leptin (β ≈ −0.4, 95% CrI: −0.51 to −0.28) remained consistent across all models, with Rˆ = 1.00 for all parameters. These findings reinforce the robustness of the primary results and indicate that the observed effects are not sensitive to prior specification or sampling variability.

## Discussion

### Main findings

This cross-sectional study examined links between circulating oxytocin, leptin, metabolic dysfunction, and disordered-eating behaviors across metabolic obesity phenotypes, with all primary models adjusted for age and sex and sex×oxytocin interactions tested. The central finding is a robust inverse association between plasma oxytocin and eating-disorder severity (EDE-Q global, r = –0.734, p < 0.001), alongside consistent positive associations for leptin and adiposity indices. Allowing for non-linearity, spline modeling suggested a risk nadir above ~90 pg/mL; below ~90.5 pg/mL the model’s predicted probability of EDE-Q ≥ 2.5 exceeded 50%. This operating point is exploratory and not a clinical cut-off.

### Construct validity of the proposed model

To establish construct validity, we examined interrelationships among hormonal, metabolic, and behavioral indicators. HOMA-IR and the hepatic steatosis index (HSI) showed a moderate positive correlation (r ≈ 0.52, p < 0.001), consistent with previous reports linking insulin resistance to NAFLD risk in obesity [13.14]. Both indices were markedly elevated in metabolically unhealthy phenotypes, particularly MUO compared with MHNW (median HSI difference +15.4, Cohen’s d = 1.2; p < 0.01). NAFLD-risk prevalence (HSI ≥ 36) reached ~78% in MUO versus ~11% in MHNW (p < 0.001). Importantly, HSI correlated with disordered-eating severity (r = 0.41 for EDE-Q global, p < 0.01), indicating that behavioral pathology parallels metabolic deterioration.

These convergent associations support the validity of our phenotype classifications and confirm that the measured constructs behave as theoretically expected—oxytocin levels decline, leptin and metabolic-risk indices rise, and eating-disorder symptomatology intensifies across worsening metabolic states. The persistence of these associations after adjustment for age, sex, and BMI further demonstrates discriminant validity, suggesting that oxytocin reflects neurobehavioral regulation beyond adiposity alone. Moreover, this internal consistency enhances the reliability of model inputs and contributes to superior discrimination (ΔAUC = 0.11) and net clinical benefit in decision-curve analysis, underscoring incremental validity of the combined oxytocin–leptin model for metabolicbehavioral risk stratification.

### Integration with existing literature

The positive association of leptin with disordered-eating indices is compatible with leptin resistance in obesity, where elevated leptin fails to suppress appetite and reward-driven intake (15). Experimental work indicates that oxytocin can reduce hedonic food intake, dampen reward-related neural responses, and enhance cognitive control in obesity and binge-eating disorder (7, 9, 10), aligning with our inverse oxytocin–EDE-Q association. Context-dependence may help explain heterogeneity in metabolic studies: population-based data have linked oxytocin positively with components of the metabolic syndrome, whereas inverse associations appear in dysglycemic or high-risk subgroups (16, 17). Such discrepancies suggest effects that vary with metabolic state, sex, and co-regulatory hormones (10). In our data, no consistent sex×oxytocin interaction was detected.

### Interpretation of the non-linear pattern and predictive modeling

The oxytocin–risk curve showed a non-monotonic (U-shaped) profile: risk was highest at low concentrations, minimized around ~90–100 pg/mL, and rose again at extreme values amid wide uncertainty bands. Mechanistically, very low oxytocin may reflect impaired synthesis/release, whereas unusually high peripheral levels could represent compensatory secretion to chronic metabolic or psychosocial stress; both remain hypotheses requiring mechanistic work. From a prediction standpoint, flexible specification and multivariable context clearly helped: in leakage-free nested cross-validation, an oxytocin-only spline model achieved AUC ~0.87 (95% CI 0.76–0.95) with mean Brier ~0.10, while a combined model (oxytocin spline + leptin, BMI, waist circumference, HSI, VAI, and a PCA-derived EDE-Q component) reached AUC ~0.97 (95% CI 0.90–1.00) with Brier ~0.05. A paired bootstrap on out-of-fold predictions showed ΔAUC ≈ 0.11 (95% CI 0.01–0.22; p≈0.02) favoring the combined model. Calibration was acceptable for the oxytocin-only model (recalibration intercept ~0.09; slope ~1.03) and suggested the combined model would benefit from recalibration prior to deployment (intercept ~−0.95; slope ~1.87). Decision-curve analysis showed greater net benefit for multivariable models across clinically relevant thresholds, supporting potential utility for triage or referral.

### Potential mechanisms

Oxytocin’s protective association against disordered eating may be mediated by actions on hypothalamic and mesolimbic circuits governing appetite and reward (7, 18). Experimental data show blunted activity in reward-related regions to palatable cues after oxytocin administration (19, 20). Interactions with leptin signaling are plausible given convergent hypothalamic pathways regulating energy balance (10). Beyond central effects, modulation of the gut–brain axis is a candidate mechanism: *Lactobacillus reuteri* has been shown to increase endogenous oxytocin and improve metabolic parameters (21). Whether such approaches could shift oxytocin into a lower-risk range and reduce disordered-eating risk remains to be tested.

### Strengths and limitations

Strengths include phenotype-stratified analyses, rigorous control for confounding, flexible modeling of oxytocin, and leakage-free nested cross-validation with bootstrap uncertainty, calibration assessment, and decision-curve analysis. However, several important limitations must be acknowledged.

First, the cross-sectional design precludes causal inference; observed associations may reflect reverse causality or unmeasured confounding. Second, plasma oxytocin, while measured under standardized conditions, may not accurately represent central oxytocin activity due to blood–brain barrier dynamics and known assay variability, particularly in ELISA measurements (22). Third, the modest, geographically localized sample limits generalizability and increases the risk of model overfitting, especially given the high number of predictors relative to sample size. Small subgroup sizes (e.g., MHNW n = 18, MUNW n = 12, MUOW n = 13) further reduced statistical power for between-group comparisons. The MHNW group’s BMI (median 23.8, IQR 23.4–24.8) clustered near the upper normal-weight range, potentially biasing comparisons with metabolically unhealthy phenotypes. In addition, the predominance of female participants (76.8%) may reduce generalizability to males. Nevertheless, Bayesian sensitivity analyses employing alternative prior distributions and likelihood functions yielded nearly identical posterior estimates, confirming the stability of the main effects despite these constraints (**Supplementary Table S4**). Fourth, while we adjusted for age and sex, and tested sex × oxytocin interaction, menstrual phase, hormonal contraceptive use, and other endocrine modifiers were not available and are noted as limitations. Finally, lifestyle factors (medications such as metformin and GLP-1 receptor agonists, smoking, alcohol, sleep) were only partially available and thus could not be included consistently in all models. The proposed oxytocin threshold of approximately 90.5 pg/mL was derived statistically from the present dataset and has not been externally validated; it should thus be interpreted as exploratory and hypothesis-generating rather than clinically definitive.

### Clinical and research implications

Oxytocin appears informative but is most useful when combined with metabolic and behavioral context. Our multivariable model improved discrimination and net benefit over oxytocin alone, suggesting a path toward pragmatic risk stratification in obesity care. Future work should prioritize (i) external validation and calibration transfer, (ii) mechanistic studies of the non-linear association, including potential sex-specific effects, and (iii) interventional studies to test whether modifying oxytocin-related pathways (pharmacologic or microbiome-targeted) can reduce disordered-eating severity and improve metabolic outcomes.

### Future directions

Longitudinal studies should evaluate whether baseline oxytocin predicts the onset or persistence of disordered eating and whether interventions—pharmacological, behavioral, or microbiome-targeted— can modify oxytocin trajectories and clinical outcomes. Randomized controlled trials of intranasal oxytocin in metabolic obesity should incorporate metabolic phenotype stratification, sex-specific analyses, and neuroimaging to clarify central mechanisms. Validation of non-linear associations in larger, more diverse cohorts will be essential before clinical thresholds can be proposed.

## Conclusion

Our findings suggest that circulating oxytocin, particularly when modeled non-linearly and adjusted for key confounders, is associated with disordered eating severity across metabolic obesity phenotypes. The identification of an approximate 90.5 pg/mL threshold offers a hypothesis-generating insight into potential biomarker use, but its clinical application awaits external validation. Integrating oxytocin with metabolic and behavioral markers could enhance early identification of high-risk individuals and inform personalized intervention strategies.

## Data Availability

Data are available from the corresponding author upon reasonable request.
