# Supplementary material for: Plasma oxytocin and leptin in relation to disordered eating: evidence from non-linear modeling across metabolic obesity phenotypes
Source: Front Endocrinol (Lausanne). 2025 Nov 20;16:1693509. doi: 10.3389/fendo.2025.1693509 (PMC12675231; doi:10.3389/fendo.2025.1693509)

Supplementary table 1. STROBE Checklist for Cross-Sectional Studies

This checklist follows the Strengthening the Reporting of Observational Studies in Epidemiology (STROBE) guidelines. It summarizes how each recommendation has been addressed in the manuscript entitled "PLASMA OXYTOCIN AND LEPTIN IN RELATION TO DISORDERED EATING: EVIDENCE FROM NON-LINEAR MODELING ACROSS METABOLIC OBESITY PHENOTYPES."

Item No.	Section / Topic	STROBE Recommendation	Reported on Page / Comments
1	Title & Abstract	Indicate study design and objectives in title or abstract	Title and abstract specify cross-sectional design and summarize objectives.
2	Background / Rationale	Explain scientific background and rationale	Introduction – obesity, oxytocin, leptin, and eating behavior context.
3	Objectives	State specific objectives and hypotheses	End of Introduction – aims to evaluate oxytocin/leptin as predictors of disordered eating.
4	Study Design	Present key elements of study design early	Methods – first paragraph describes cross-sectional observational design.
5	Setting	Describe setting, locations, and dates	Methods – conducted at Republican Specialized Scientific-Practical Medical Center of Endocrinology, Tashkent, 2023.
6a	Participants – eligibility	Give eligibility criteria and methods of selection	Methods – inclusion/exclusion criteria defined for all participants.
6b	Participants – numbers	Describe number of participants at each stage	Methods + Results – n = 99; Table 1 shows demographics.
7	Variables	Define all outcomes, exposures, predictors, confounders	Methods – describes all metabolic, hormonal, and behavioral variables.
8	Data sources / Measurement	Describe sources and methods of data collection	Methods – biochemical assays and questionnaire administration described.
9	Bias	Describe efforts to address potential sources of bias	Discussion – notes sampling bias, female predominance, ELISA variability.
10	Study size	Explain how study size was determined	Methods – exploratory, unfunded study with pragmatic sample (n=99).

11	Quantitative variables	Explain how quantitative variables were handled in analyses	Methods – scaling, transformations, spline terms for oxytocin, PCA for EDE-Q subscales.
12a	Statistical methods	Describe all statistical methods, including confounder control	Methods – Kruskal–Wallis, regression models, nested CV, Bayesian analysis.
12b	Subgroups / interactions	Describe any subgroup analyses and interactions	Results – phenotype-specific group comparisons (MHNW, MUNW, MUOW, MUO).
12c	Missing data	Explain how missing data were addressed	Methods – imputation within cross-validation folds.
12d	Sensitivity analyses	Describe any sensitivity analyses	Supplementary – Bayesian hierarchical sensitivity analysis (Tables S3–S4).
13a	Participants analyzed	Report numbers in each analysis	Results – n = 99; group sizes provided in all tables.
14	Descriptive data	Give characteristics of study participants	Table 1 – baseline demographic and biochemical characteristics.
15	Outcome data	Report numbers of outcome events or summary measures	Tables 2–4 – prevalence and associations of disordered eating measures.
16a	Main results	Present unadjusted and adjusted estimates with precision	Tables 5–7 – regression models with coefficients, 95% CI, and p-values.
16b	Category boundaries	Report category boundaries when continuous variables categorized	Phenotype cut-offs defined in Methods and Supplementary Table S1.
17	Other analyses	Report other analyses done (e.g. subgroups, interactions)	Supplementary – Bayesian and decision-curve analyses described.
18	Key results	Summarize key results with reference to objectives	Discussion – reiterates inverse oxytocin–leptin association and predictive value.
19	Limitations	Discuss limitations of the study	Discussion – covers sample size, female predominance, cross-sectional design, ELISA variability.
20	Interpretation	Give cautious overall interpretation of results	Final Discussion – emphasizes exploratory interpretation and clinical relevance.

21	Generalisability	Discuss the generalisability of the study results	Discussion – limited external validity due to sex imbalance and sample size.
22	Funding	Give the source of funding and the role of funders	Funding – unfunded, investigator-initiated study; no external sponsors.

Supplementary methods

Phenotyping definitions

Participants were stratified into four metabolic obesity phenotypes based on the following cut-offs:

- **Metabolically Healthy Normal Weight (MHNW)**: BMI 18.5–24.9 kg/m², HOMA-IR < 2.5, HSI < 30.
- **Metabolically Unhealthy Normal Weight (MUNW)**: BMI 18.5–24.9 kg/m², HOMA-IR ≥ 2.5 and/or HSI ≥ 30.
- **Metabolically Unhealthy Overweight (MUOW)**: BMI 25.0–29.9 kg/m², HOMA-IR ≥ 2.5 and/or HSI ≥ 30.
- **Metabolically Unhealthy Obese (MUO)**: BMI ≥ 30.0 kg/m², HOMA-IR ≥ 2.5 and/or HSI ≥ 30.

These cut-offs were selected to reflect metabolic health status and align with established literature [Blüher, 2020; Lee et al., 2010].

Assay protocols

Oxytocin was quantified using a competitive ELISA kit (Catalog No. EN-E-EL-0029, Elabscience®, Wuhan, China; sensitivity 4.69 pg/mL; detection range 7.81–500 pg/mL). Plasma samples underwent solid-phase extraction (SPE) using C18 cartridges (Waters, USA): 1 mL plasma was acidified with 1% trifluoroacetic acid (TFA), loaded onto preconditioned C18 columns, washed with 0.1% TFA, and eluted with 60% acetonitrile in 0.1% TFA. Eluates were evaporated under nitrogen and reconstituted in assay buffer. Leptin was measured using a sandwich ELISA kit (Catalog No. E-EL-H6017, Elabscience®; sensitivity 9.38 pg/mL; detection range 15.63–1000 pg/mL), with samples diluted 1:50. Intra- and inter-assay coefficients of variation (CVs) were <10% and <12% for oxytocin, and <10% for leptin. Calibration used a four-parameter logistic model. Pooled plasma controls confirmed no batch effects.

Statistical procedures

Indices calculated:

- **HOMA-IR**: [Fasting Insulin (μU/mL) × Fasting Glucose (mmol/L)] / 22.5 [Matthews et al., 1985].
- **HSI**: 8 × (ALT/AST) + BMI (+2 if female; +2 if diabetic); HSI ≥ 36 indicates high NAFLD probability, <30 low, 30–36 gray zone [Lee et al., 2010].
- **VAI** (female): [WC / (36.58 + 1.89 × BMI)] × (TG / 0.81) × (1.52 / HDL); (male): [WC / (39.68 + 1.88 × BMI)] × (TG / 1.03) × (1.31 / HDL) [Amato et al., 2010].
- **Atherogenic Index**: log10(TG/HDL-C).

Regression models:

Model A (HOMA-IR): OLS with HC3 robust SEs; predictors included leptin, BMI, restrained eating (RE), sweet eating (SE); backward elimination ($p < 0.10$); $R^2 = 0.52$, all VIF < 5.

- **Model B (Global EDE-Q)**: OLS with HC3 robust SEs; predictors included leptin, emotional eating (EE), external eating (EX), food addiction (FA), night eating (NE), binge eating (BE), sweet eating (SE); backward elimination ($p < 0.10$); $R^2 = 0.904$, max VIF ≈ 8.46 for EX. Sensitivity analysis used PCA-derived EDE-Q component (PC1) to reduce collinearity (max VIF ≈ 4.9).

Predictive modeling:

- **Nested Cross-Validation**: Outer 5-fold, inner 5-fold. Preprocessing (median/mode imputation, standardization, oxytocin spline basis [df = 4, knots at empirical quantiles], PCA of EDE-Q subscales [PC1 retained]) performed within inner folds to prevent leakage.
- **Elastic-Net Logistic Model**: Hyperparameters tuned via inner-fold AUC (C in {0.1, 0.3, 1, 3, 10}; l1_ratio in {0, 0.25, 0.5, 0.75, 1}). Combined model included oxytocin spline, leptin, BMI, WC, HSI, VAI, PCA-EDE-Q.
- **Performance Metrics**: Out-of-fold (OOF) ROC AUC, Brier score, bootstrap 95% CIs (B = 2,000) for AUC, sensitivity, specificity, PPV, NPV, Youden-optimal thresholds. Paired bootstrap for ΔAUC. Logistic recalibration (intercept, slope) on OOF predictions. Decision-curve analysis computed net benefit for threshold probabilities 0.10–0.80.

Handling missing data: Missingness summarized by variable class; within-fold median (continuous) or mode (categorical) imputation applied before scaling.

Supplementary results

1. Extended post hoc comparisons between metabolic phenotypes

Detailed pairwise comparisons for disordered eating subscales between metabolic obesity phenotypes are shown in **Supplementary Table S2**. After correction for multiple testing (FDR-adjusted Dunn's tests), individuals with metabolically unhealthy obesity (MUO) exhibited significantly higher scores across most *EDE-Q* and *EBA-O* domains compared with metabolically healthy normal-weight (MHNW) and metabolically unhealthy normal-weight (MUNW) groups ($p < 0.01$). The strongest group differences were observed for weight concern, shape concern, eating concern, and binge eating, whereas differences between MHNW and MUNW were not significant.

These results corroborate the main findings (Figure 2, Table 3 in the main text), emphasizing that metabolic unhealthiness and obesity severity jointly associate with more pathological eating behaviors.

Supplementary Table 2 . Post hoc pairwise comparisons of disordered eating subscales between obesity phenotypes.

**Subscale**	**MHNW vs MUNW**	**MHNW vs MUO**	**MHNW vs MUOW**	**MUNW vs MUO**	**MUNW vs MUOW**	**MUO vs MUOW**
**WC_Score**	1.000	**3.59×10^{-11}**	0.168	**4.59×10^{-6}**	1.000	**0.00329**
**SC_Score**	1.000	**2.31×10^{-7}**	0.0769	**0.00236**	1.000	0.348

Subscale	MHNW vs MUNW	MHNW vs MUO	MHNW vs MUOW	MUNW vs MUO	MUNW vs MUOW	MUO vs MUOW
EC_Score	1.000	**4.25×10^{-10}**	0.130	**2.75×10^{-5}**	1.000	**0.0150**
DR_Score	1.000	**0.00501**	**0.0477**	0.544	0.807	1.000
BE score	1.000	**1.80×10^{-5}**	0.185	**0.0163**	1.000	0.714
HP score	1.000	**1.66×10^{-8}**	0.0512	**0.000303**	0.672	0.203
FA score	1.000	**3.00×10^{-6}**	0.192	**0.00306**	1.000	0.338
NE score	1.000	**9.80×10^{-5}**	0.516	**0.01998**	1.000	0.467
RE score	1.000	**1.00×10^{-6}**	**0.0202**	**0.00741**	0.539	1.000
EX score	1.000	**2.22×10^{-10}**	0.169	**1.94×10^{-5}**	1.000	**0.00750**
Global_EDEQ	1.000	**2.70×10^{-9}**	**0.0184**	**0.000285**	0.529	0.282

Note: Values are p-values from Dunn's post hoc test with Bonferroni correction. Significant results after false discovery rate (FDR) control ($p < 0.05$) are shown in **bold**. MHNW — metabolically healthy normal weight; MUNW — metabolically unhealthy normal weight; MUOW — metabolically unhealthy overweight; MUO — metabolically unhealthy obese.

2. Predictive modeling performance details

Performance metrics from nested 5×5 cross-validation for all predictive models are presented in **Supplementary Tables S3–S4**. The oxytocin-only spline model achieved a mean AUC of **0.865 ± 0.081** (bootstrap 95% CI 0.756–0.947) and mean Brier score **0.103**, with an optimal probability threshold corresponding to ~90.5 pg/mL oxytocin.

The combined multivariable elastic-net model (oxytocin spline, leptin, BMI, WC, HSI, VAI, EDE-Q component) achieved **AUC 0.969 ± 0.046** (95% CI 0.897–1.000) and mean Brier score **0.045**, with calibration intercept −0.95 (95% CI −2.74 to 0.83) and slope 1.87 (95% CI 0.84–2.90).

Paired bootstrap analysis confirmed a significant improvement versus the oxytocin-only model (**ΔAUC = 0.114**, 95% CI 0.014–0.217; *p* = 0.024). Random Forest and Gradient Boosting achieved similar discrimination (AUCs ~0.96–0.99) but displayed poorer calibration (slopes ≈0.5–1.3).

These findings are consistent with the main text (Figures 4–6), demonstrating that oxytocin has predictive value for disordered eating, but the combined metabolic-behavioral model provides superior calibration and clinical utility.

Supplementary table 3. Predictive performance for EDE-Q ≥ 2.5 from nested 5×5 cross-validation (OOF predictions)

Model	AUC (mean ± SD)	AUC 95% CI (OOF bootstrap)	Brier (mean)	Calibration intercept (95% CI)	Calibration slope (95% CI)	Youden p̂*	Sensitivity (95% CI)	Specificity (95% CI)

**Oxytocin-only (spline)**	**0.865 ± 0.081**	**0.756–0.947**	**0.103**	0.09 (−0.55, 0.73)	1.03 (0.67, 1.38)	0.69	0.94 (0.87–0.99)	0.83 (0.70–0.95)
**Combined (Elastic Net)**	**0.969 ± 0.046**	**0.897–1.000**	**0.045**	−0.95 (−2.74, 0.83)	1.87 (0.84, 2.90)	0.77	0.98 (0.95–1.00)	0.92 (0.82–1.00)
**Random Forest**	**0.996 ± 0.009**	—	**0.029**	−0.29 (−1.64, 1.07)	1.27 (0.58, 1.95)	—	—	—
**Gradient Boosting**	**0.959 ± 0.028**	—	**0.064**	0.36 (−0.25, 0.98)	0.51 (0.19, 0.83)	—	—	—

All numbers are from leakage-free nested cross-validation (outer 5-fold; inner 5-fold). Preprocessing (imputation, scaling, spline basis for oxytocin, PCA of EDE-Q subscales) and tuning were performed within folds. AUC CIs are bootstrap CIs computed from OOF predictions. Brier is the mean across outer folds. Intercept/slope are from logistic recalibration on OOF predictions. Youden $\hat{p}$* is the OOF probability threshold maximizing (sensitivity + specificity−1). Although AUCs were similar across models, calibration intercept/slope favored the combined elastic-net model over Gradient Boosting (over-confident; slope ≈0.51) and was comparable to Random Forest.

Supplementary Table 4. Paired bootstrap ΔAUC (OOF predictions, 2,000 resamples)

Contrast	ΔAUC	95% CI	p-value
**Combined − Oxytocin-only**	**0.114**	**0.014–0.217**	**0.024**
Random Forest − Combined	0.005	−0.003–0.021	0.749
Gradient Boosting − Combined	−0.011	−0.060–0.042	0.654
Random Forest − Gradient Boosting	0.016	−0.026–0.060	0.470

3. Bayesian hierarchical sensitivity analysis

To evaluate the robustness of frequentist results under small-sample uncertainty, a Bayesian hierarchical sensitivity analysis was performed across multiple prior and likelihood specifications. Posterior estimates for oxytocin and leptin remained stable across all scenarios, and all models showed excellent convergence ($\hat{R}$ = 1.00).

Posterior means for oxytocin were approximately β ≈ 0.9 (95% CrI: 0.75–1.04) and for leptin β ≈ −0.4 (95% CrI: −0.51 to −0.28), closely mirroring the coefficients obtained from the frequentist regression. No scenario (weaker or stronger priors, Student-t likelihood, or fixed-effects variant) materially altered inference direction or significance. These results confirm that the observed associations between oxytocin, leptin, and eating-behavior outcomes were robust to prior specification, likelihood form, and hierarchical structure.

Model comparison using the Watanabe–Akaike Information Criterion (WAIC) showed minimal differences between models (Supplementary Figure S1), supporting comparable predictive adequacy across sensitivity conditions. Posterior predictive checks demonstrated good agreement between observed and simulated data (Supplementary Figure S2), and MCMC trace plots confirmed chain stability and convergence across scenarios (Supplementary Figure S3).

Supplementary Table S4 summarizes posterior means, credible intervals, and $\hat{R}$ values for key parameters. These results confirm that the main associations between oxytocin, leptin, and eating-behavior outcomes are robust to prior specification and sampling variability.

Supplementary Table 4. Bayesian hierarchical sensitivity analysis across prior and likelihood scenarios.

Scenario	Parameter	Posterior Mean	95% CrI (Lower–Upper)	$\hat{R}$
Main Model	x1 (Oxytocin)	0.914	0.796 – 1.036	1.00
	x2 (Leptin)	−0.404	−0.509 – −0.283	1.00
Weaker Priors	x1 (Oxytocin)	0.917	0.791 – 1.035	1.00
	x2 (Leptin)	−0.404	−0.514 – −0.288	1.00
Stronger Priors	x1 (Oxytocin)	0.871	0.754 – 0.986	1.00
	x2 (Leptin)	−0.390	−0.499 – −0.276	1.00
Student-t Likelihood	x1 (Oxytocin)	0.907	0.788 – 1.024	1.00
	x2 (Leptin)	−0.400	−0.505 – −0.293	1.00
Fixed-Effects Only	Intercept	0.045	−0.060 – 0.154	1.00
	g_coef[0]	0.125	−0.071 – 0.313	1.00
	g_coef[1]	−0.009	−0.193 – 0.183	1.00
	g_coef[2]	−0.139	−0.321 – 0.046	1.00
	g_coef[3]	0.023	−0.161 – 0.204	1.00
	x1 (Oxytocin)	0.920	0.798 – 1.039	1.00
	x2 (Leptin)	−0.401	−0.521 – −0.294	1.00
	σ_γ (Residual SD)	0.543	0.465 – 0.621	1.00

Notes: Posterior means and 95% credible intervals (CrI) are derived from hierarchical Bayesian regression models under different prior and likelihood specifications. All models showed excellent convergence ($\hat{R}$ = 1.00). Stable posterior estimates for oxytocin and leptin across scenarios indicate that the main associations were robust to prior strength, likelihood choice, and random-effect structure.

Across all sensitivity scenarios, posterior means for oxytocin (~0.9) and leptin (~−0.4) remained consistent, confirming that the main associations between hormonal predictors and eating-behavior outcomes were robust to prior specification and model formulation. These Bayesian results reinforce the stability of the frequentist findings presented in the main text.

Supplementary Figure S1. Model comparison by WAIC across Bayesian sensitivity scenarios. Lower WAIC indicates better expected out-of-sample fit; all models demonstrated nearly identical WAIC values.

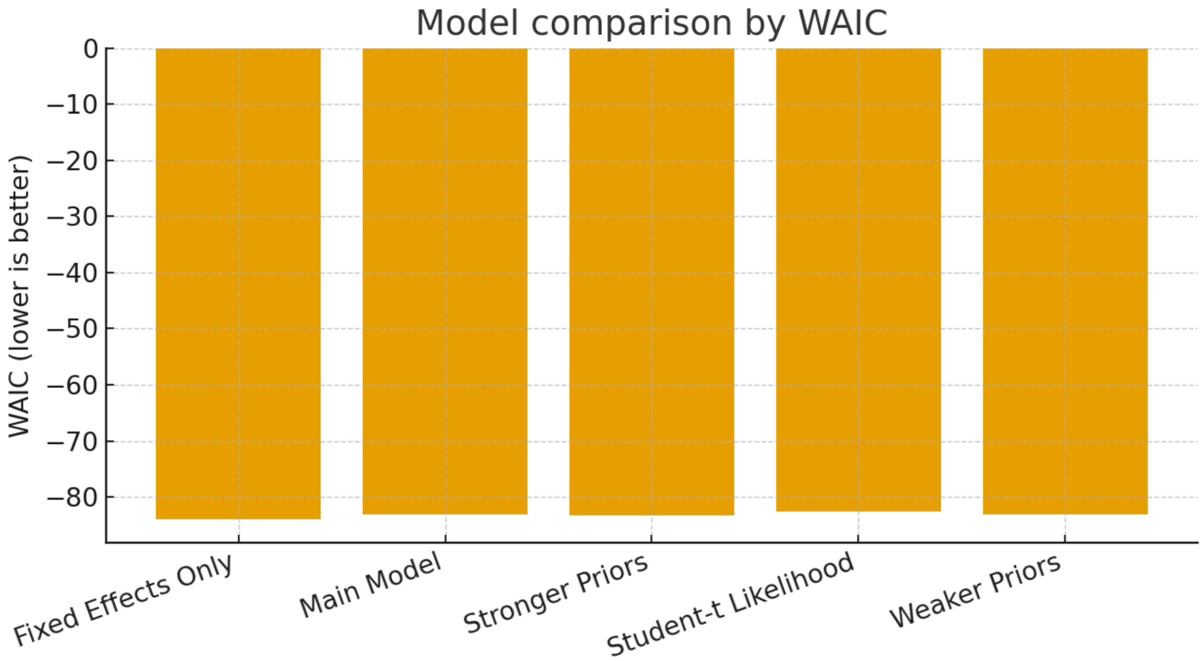

Supplementary Figure S2. Posterior predictive check for the main hierarchical model showing close alignment between observed and simulated distributions.

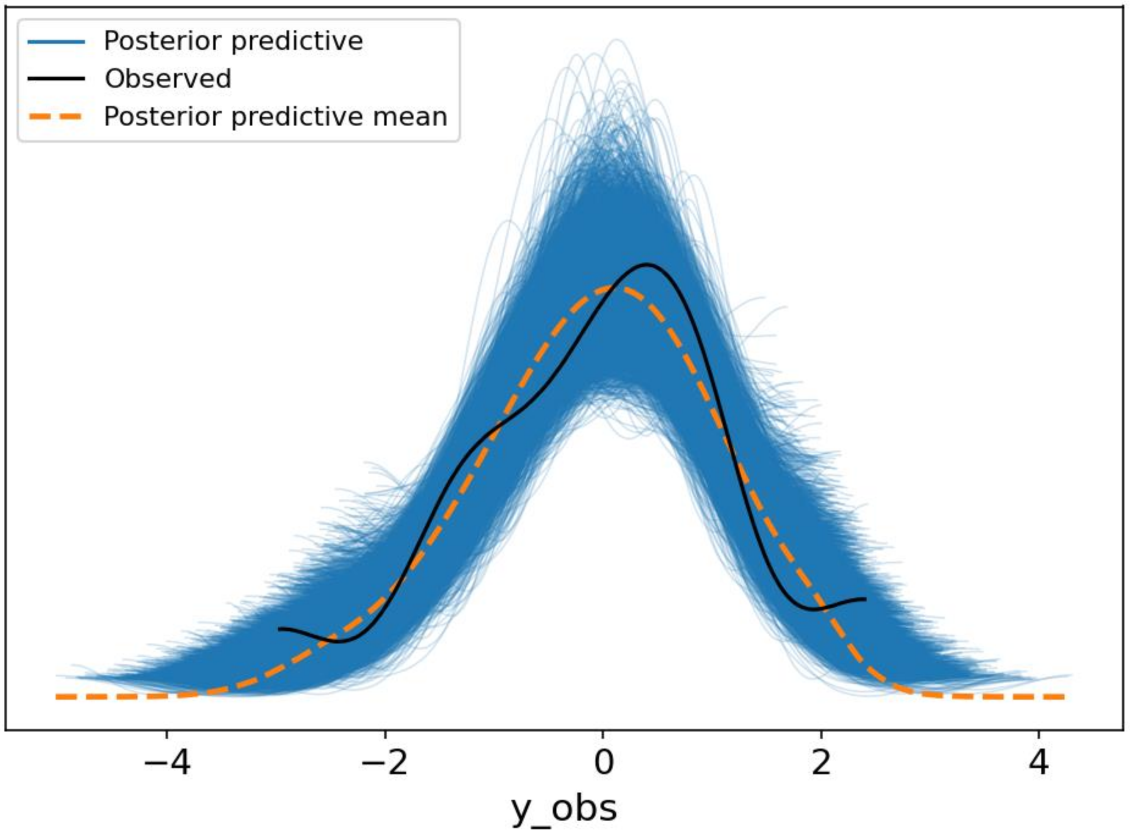

Supplementary Figure S3. Posterior densities and MCMC trace plots for oxytocin (β_1) and leptin (β_2) under the main and Student-t likelihood models, confirming convergence and consistency across sampling conditions.

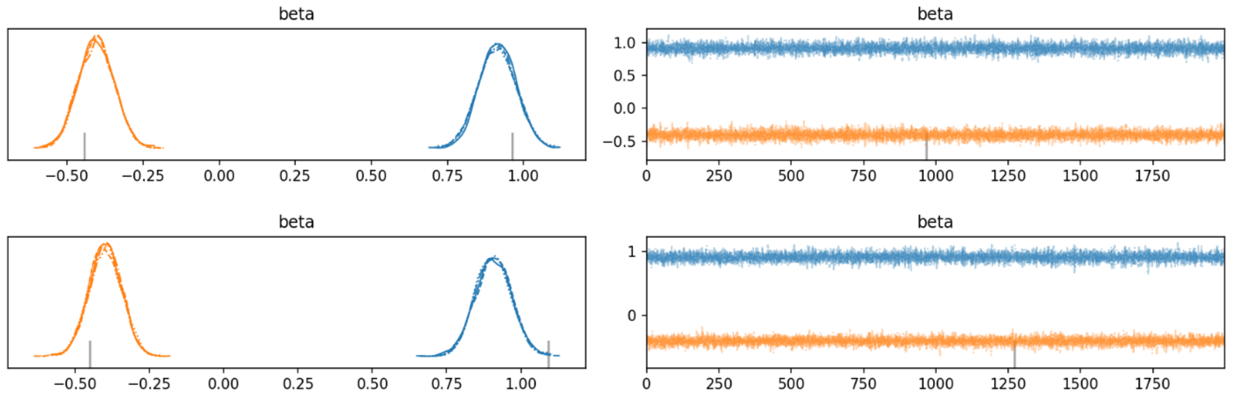

Supplement: Supplementary file 1 [file DataSheet1.pdf]
